# Post-Processing Optimization of MDLP-Fabricated 316L Stainless Steel: Microstructural Evolution and Mechanical Properties

**DOI:** 10.3390/ma19091769

**Published:** 2026-04-27

**Authors:** Zequn Wu, Weiwei Liu, Hongzhi Zhou, Xing Zhang, Yao Chen, Qinghao Zhang, Wenjie Xu, Wenli Li, Zhanwen Xing

**Affiliations:** 1 School of Mechanical and Electrical Engineering, Soochow University, Suzhou 215021, Chinaxingzhang@suda.edu.cn (X.Z.); chenyao@suda.edu.cn (Y.C.);; 2 College of Mechanical Engineering, Hunan Institute of Science and Technology, Yueyang 414015, China

**Keywords:** MDLP, 316L stainless steel, post-processing, decarburization, sintering

## Abstract

Metal Digital light processing (MDLP) offers high resolution and excellent surface quality, but the final properties of printed parts are highly dependent on post-processing. In this study, the effects of debinding, decarburization, and sintering on the shape fidelity, microstructure, and mechanical properties of MDLP-fabricated 316L stainless steel were systematically investigated. The optimal post-processing route consisted of debinding in an inert atmosphere, decarburization in air within 400–600 °C, and sintering at 1370 °C for 4 h under flowing nitrogen. Under these conditions, the sintered parts achieved a relative density of 98.03 ± 0.23%, hardness of 380.63 ± 9.15 HV, elastic modulus of 213.47 ± 5.5 GPa, tensile strength of 519.7 ± 22 MPa, and elongation at fracture of 76.8 ± 9.3%. Microstructural analysis showed that increasing the sintering temperature reduced porosity and smoothed the morphology of Cr-rich oxygen-containing second phase regions, thereby alleviating stress concentration and improving mechanical properties. This study provides an effective post-processing strategy for MDLP-fabricated 316L stainless steel and examines the microstructural origins of the observed property evolution.

## 1. Introduction

Additive manufacturing (AM) has attracted increasing attention in metal manufacturing because of its ability to fabricate geometrically complex components with high material utilization and design flexibility [1,2,3]. Among the various AM technologies, metal digital light processing (MDLP) has emerged as a promising route for producing fine-featured metal parts with high resolution and good surface quality. In MDLP, a photocurable metal paste is selectively cured in a layer-by-layer manner and subsequently transformed into dense metallic components through thermal post-processing. These characteristics give MDLP considerable potential for the fabrication of precision components such as medical devices, micro heat sinks, decorative parts, and watch components [4,5]. Recent studies have demonstrated the feasibility of MDLP for stainless steel and other metallic materials [4,6,7,8,9].

Compared with fusion-based metal AM processes [10,11,12,13], MDLP avoids rapid melting and solidification during fabrication, thereby reducing defects associated with thermal stress and enabling higher geometrical fidelity. However, the advantages offered by the printing process do not directly guarantee the quality of the final parts. Unlike laser-based processes that produce near-dense metallic structures in a single step, MDLP-fabricated green bodies contain a high fraction of organic components and require subsequent debinding and sintering to achieve densification. As a result, the final dimensional accuracy, microstructure, and mechanical properties are highly dependent on post-processing. In particular, the removal of organics, elimination of residual carbon, control of oxidation, and sintering conditions must be carefully coordinated to avoid cracking, distortion, abnormal melting, and insufficient densification.

For 316L stainless steel (316L SS), these post-processing issues are particularly important. This alloy is widely used because of its excellent corrosion resistance, good ductility, and balanced mechanical properties, making it attractive for precision applications [8,14,15,16,17]. Nevertheless, in MDLP-fabricated 316L SS, the high organic content of the photocurable paste and the porous structure generated after debinding make the material highly sensitive to the atmosphere and temperature during thermal treatment. If debinding is too aggressive, rapid decomposition of the organic components may damage the particle skeleton and induce cracking. If residual carbon is not adequately removed, it may lower the local melting point during sintering and cause deformation or collapse. Meanwhile, exposure to oxygen during thermal treatment may accelerate oxidation because the debound body possesses a highly porous structure and a large specific surface area, which in turn affects neck growth, densification behavior, and second-phase evolution. Therefore, the post-processing route for MDLP-fabricated 316L SS cannot be directly adopted from conventional powder metallurgy, metal injection molding (MIM), or binder jetting (BJ), although these processes provide useful references [18,19,20,21].

Existing studies on MDLP-fabricated 316L SS have mainly focused on slurry formulation, printability, and the general optimization of sintering conditions [4,8,22,23,24,25]. However, a systematic understanding of the overall post-processing route remains lacking. In particular, the individual and combined effects of debinding atmosphere, decarburization conditions, and sintering parameters on shape fidelity, densification, microstructural evolution, and mechanical properties have not been fully clarified. More importantly, the formation and evolution of oxygen-rich phases during post-processing, as well as their interactions with pores and their influence on fracture behavior, remain insufficiently understood. This knowledge gap limits the mechanistic understanding of defect formation and hinders the rational optimization of post-processing parameters for MDLP-fabricated 316L SS components.

In this study, the post-processing route for MDLP-fabricated 316L SS was systematically investigated, with emphasis on debinding, decarburization, and sintering. The effects of these parameters on dimensional stability, relative density, microstructure, and mechanical properties were evaluated, and the underlying mechanisms governing property evolution were analyzed. An optimized post-processing route was identified, consisting of debinding in an inert atmosphere, decarburization in air at 400–600 °C, and sintering at 1370 °C for 4 h under flowing nitrogen. The significance of this work is twofold: from a practical perspective, it establishes a reliable processing window that enables the reproducible fabrication of near-dense, crack-free 316L SS components using MDLP; from a scientific perspective, it examines the influence of residual carbon removal and oxygen-rich phase evolution on the final microstructure and mechanical behavior of MDLP-processed 316L SS, thereby providing insight into the mechanistic basis for future post-processing optimization of similar vat photopolymerization-based metal AM systems.

## 2. Materials and Methods

### 2.1. Preparation of 316L SS Photocurable Paste and MDLP Printing

Micrometer-sized 316L SS powder (Zhongyuan Advanced Materials, Ningbo, China) was used as the raw material for preparing the photocurable paste. The particle size distribution of the powder was D10 = 5.33 μm, D50 = 10.25 μm, and D90 = 17.24 μm, and its chemical composition is listed in Table 1. The photocurable paste was prepared by mixing the 316L SS powder with a photosensitive resin system using a planetary centrifugal mixer (RM2000C, SINOMIX Co., Ltd., Mianyang, China), yielding a metal solid loading of 50 vol%.

The photosensitive resin system consisted of 1,6-hexanediol diacrylate (HDDA) and trimethylolpropane triacrylate (TMPTA) (Sartomer Co., Ltd., Exton, PA, USA) at a mass ratio of 1:4. Diphenyl (2,4,6-trimethylbenzoyl) phosphine oxide (TPO, IGM RESINS Co., Ltd., Waalwijk, The Netherlands) was added at 5 wt% relative to the resin as the photoinitiator. In addition, BYK-111 (BYK-Chemie GmbH, Wesel, Germany) was added at 1 wt% relative to the 316L SS powder as the dispersant to reduce particle agglomeration. CRAYVALLAC LV (Arkema Co., Ltd., Paris, France) was added at 100 wt% relative to BYK-111 as the thixotropic agent to increase the yield stress of the paste and improve its storage stability.

MDLP printing was carried out using a DLP metal printer (iMLM 320, ZRapid Tech, Suzhou, China). The printing parameters were set as follows: light source power, 100 mW; exposure time, 9 s; and layer thickness, 30 μm. To improve the bottom surface quality of the printed parts and facilitate removal of the green bodies, a free-link support structure with a gap of 90 μm was adopted.

### 2.2. Thermal Post-Processing of 316L SS Green Bodies

Thermal post-processing, including debinding, decarburization, and sintering, was conducted in a tube furnace (GSL-1400X, HF-Kejing, Hefei, China). To evaluate the effectiveness of different binder-removal strategies, two distinct processing routes were compared: (i) debinding in an inert atmosphere (nitrogen or argon) followed by a separate decarburization step in air, and (ii) direct debinding in air without a subsequent decarburization step. Accordingly, depending on the experimental design, flowing nitrogen, argon, or air was used during debinding, flowing air was used during decarburization, and flowing nitrogen was used during sintering. For route (i), inert atmospheres (nitrogen and argon) were employed during debinding to permit gradual thermal decomposition of the organic binder, and a subsequent decarburization step in air was introduced to facilitate the oxidative removal of residual carbon. For route (ii), direct debinding in air was used to simultaneously remove the organic binder and oxidize residual carbon, thereby eliminating the need for a separate decarburization step. Nitrogen was selected as the sintering atmosphere for both routes as a cost-effective protective medium that prevents severe oxidation of the 316L stainless steel at elevated temperatures. The gas flow rate was fixed at 400 mL/min for all thermal treatments, controlled by a pressure-reducing valve and verified by the flow meter reading throughout the entire process.

**Figure 1:**
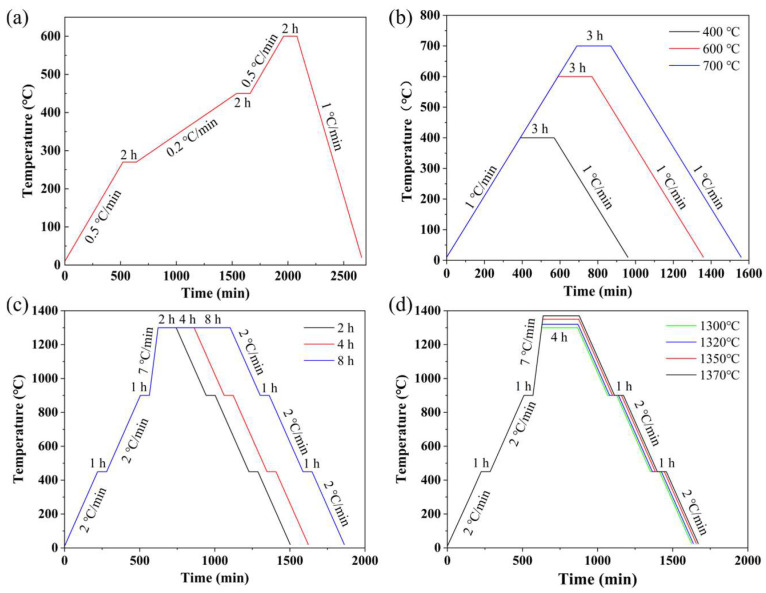
Temperature profiles used in the experiments. (**a**) Debinding test; (**b**) Decarburization test; (**c**) Sintering holding-time test; (**d**) Sintering-temperature test.

Based on preliminary experimental results, stepwise heating profiles were employed for debinding, decarburization, and sintering. After debinding, the green bodies were divided into two groups. One group was directly sintered, whereas the other group underwent an additional decarburization step before sintering. Specifically, for the debinding test, the furnace was heated from room temperature (20 °C) to 270 °C at a rate of 0.5 °C/min and held for 2 h, then heated to 450 °C at 0.2 °C/min and held for 2 h, further heated to 600 °C at 0.5 °C/min and held for 2 h, and finally cooled to room temperature at 1 °C/min. For the decarburization test, the furnace was heated from room temperature (20 °C) to 400, 600, and 700 °C at a rate of 1 °C/min, held for 3 h at each target temperature, and then cooled to room temperature at 1 °C/min. For the sintering holding-time test, the furnace was heated from room temperature (20 °C) to 900 °C at 2 °C/min, with intermediate holds of 1 h at 450 °C and 900 °C, then heated to 1300 °C at 7 °C/min and held for 2, 4, and 8 h, respectively, followed by cooling to room temperature at 2 °C/min with intermediate holds of 1 h at 900 °C and 450 °C. For the sintering-temperature test, the same stepwise heating and cooling strategy was applied, but the final sintering temperatures were set at 1300, 1320, 1350, and 1370 °C, with a uniform holding time of 4 h. The temperature programs used for the debinding, decarburization, sintering holding-time, and sintering-temperature experiments are summarized in Figure 1.

### 2.3. Microstructural and Mechanical Characterization

**Figure 2:**
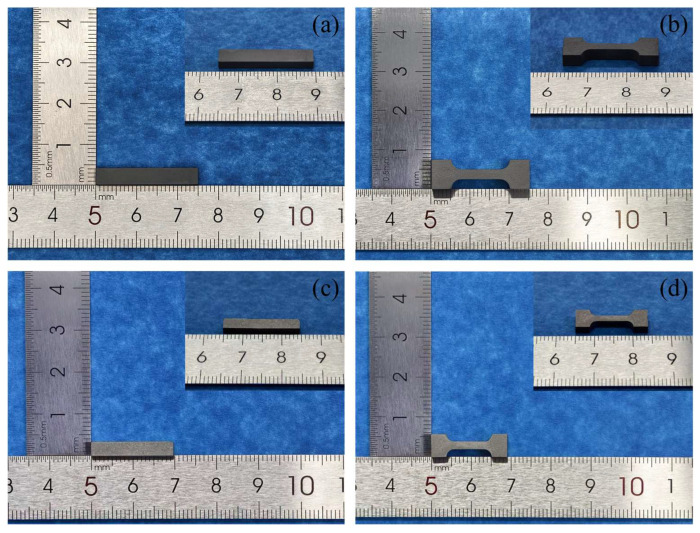
Photographs of green bodies and sintered samples. (**a**,**c**) Green body and sintered sample of rectangular bars; (**b**,**d**) Green body and sintered sample of tensile bars.

After sintering, the samples were mounted in resin, sequentially ground with 400, 800, 1200, and 2000 grit diamond papers, and then polished using a 1 μm single-crystal diamond suspension. The unetched microstructures were first examined by optical microscopy. Subsequently, the samples were etched for 5 s in a hydrochloric acid/nitric acid solution with a volumetric ratio of 3:1, and the etched microstructures and elemental distributions were characterized by scanning electron microscopy (SEM; Regulus 8230, HITACHI, Tokyo, Japan) equipped with energy-dispersive X-ray spectroscopy (EDS, Regulus 8230, HITACHI, Tokyo, Japan). Two types of specimens were used for property evaluation: rectangular bars with dimensions of 20 × 3 × 2 mm and tensile bars with a gauge length of 8 mm. Representative photographs of the green bodies and the corresponding sintered samples are presented in Figure 2. The density of the rectangular bars was measured by the Archimedes method, and the linear shrinkage was measured using a vernier caliper (K101301, Chengliang Tools Group Co., Ltd., Chengdu, China). Hardness and elastic modulus were measured using a nanoindenter (NHT2, CSM Instruments, Peseux, Switzerland) equipped with a Berkovich tip. During nanoindentation, the load was increased to 20 mN over 30 s, held for 10 s, and then unloaded at a rate of 40 mN/min. Tensile strength and elongation at fracture were measured using an electronic universal testing machine (AGS-X-10kN, SHIMADZU, Kyoto, Japan) at a crosshead speed of 1 mm/min. In addition, the fracture surfaces of the tensile specimens were characterized by SEM, and the elemental distributions on the fracture surfaces were analyzed by EDS. At least three specimens were tested for each condition, and the data are presented as mean ± standard deviation.

## 3. Results and Discussion

### 3.1. Removal of Residual Carbon After Debinding

**Figure 3:**
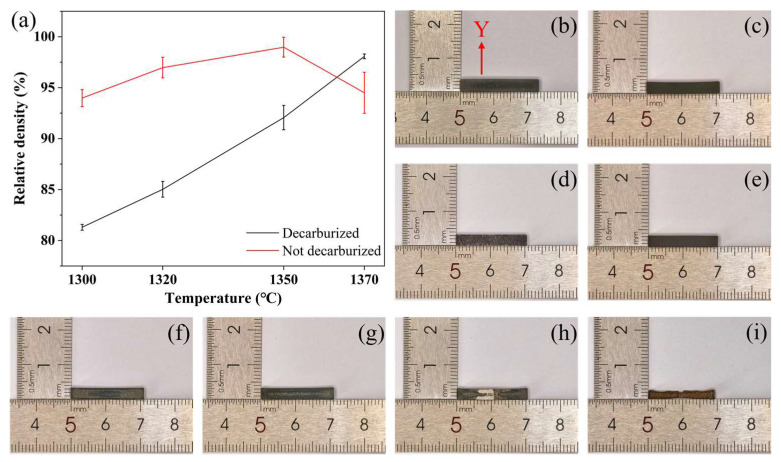
Effect of decarburization on sintered bars. (**a**) Relative density; (**b**–**e**) Top surfaces of decarburized bars sintered at 1300, 1320, 1350, 1370 °C; (**f**–**i**) Top surfaces of non-decarburized bars sintered at 1300, 1320, 1350, 1370 °C. The red arrow and letter Y indicate the width direction of the bars.

The influence of decarburization on the densification behavior and macroscopic appearance of sintered samples is summarized in Figure 3. For the specimens sintered without decarburization, severe melting and collapse were observed, particularly at higher sintering temperatures (Figure 3f–i). This behavior indicates that residual carbon originating from the 50 vol% organic phase significantly affected the subsequent sintering process. Although carbothermal reduction [26] may contribute to the apparent increase in density at relatively low temperatures (Figure 3a), the associated local reduction in melting point [18] led to unacceptable shape distortion. In contrast, the introduction of a decarburization step effectively suppressed this melting-induced deformation and improved shape retention (Figure 3b–e).

**Figure 4:**
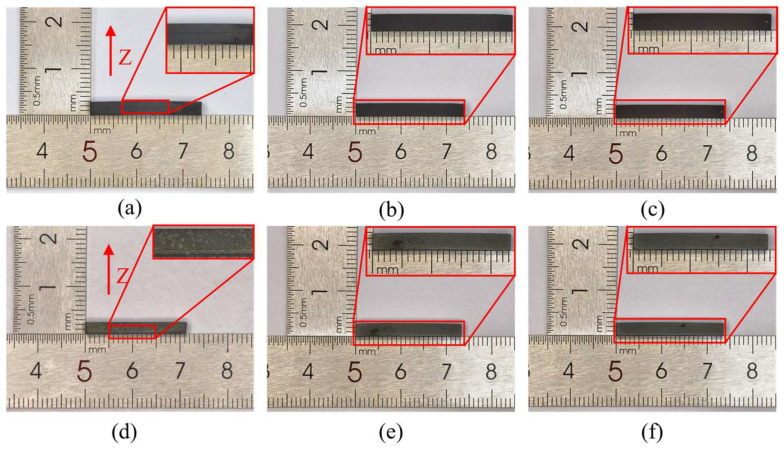
Effect of different debinding–decarburization strategies. (**a**,**d**) Green body and sintered sample after debinding in air; (**b**,**e**) Green body and sintered sample after debinding in N_2_ and decarburization in air; (**c**,**f**) Green body and sintered sample after debinding in Ar and decarburization in air. The red arrow and letter Z indicate the height direction of the bars.

To further optimize the removal of residual carbon, two processing routes were compared: direct debinding in air and debinding in an inert atmosphere followed by decarburization in air, as shown in Figure 4. When debinding was carried out in air, oxygen directly participated in the thermal decomposition of the organic components, which markedly accelerated their removal. However, this rapid decomposition also damaged the integrity of the particle skeleton, resulting in visible cracks in the debound green bodies. Although the residual carbon was largely eliminated under this condition and melting deformation during sintering was avoided, the cracks generated during debinding were retained after sintering (Figure 4a,d). In contrast, debinding in an inert atmosphere slowed the decomposition of the organic phase and preserved the structural integrity of the green body. Subsequent decarburization in air then removed the remaining carbon without inducing either cracking or local melting (Figure 4b,c,e,f). Therefore, debinding in an inert atmosphere followed by decarburization in air was identified as the preferred strategy.

**Figure 5:**
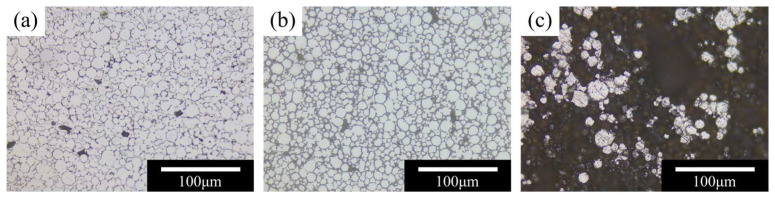
Results of decarburization temperature experiments. (**a**) 400 °C; (**b**) 600 °C; (**c**) 700 °C.

The effect of decarburization temperature on the microstructure of the debound green bodies is presented in Figure 5. At 700 °C, severe oxidation occurred inside the green body, and the optical micrographs showed a mixture of the gray-white metallic matrix and reddish-brown oxide regions. This oxidation disrupted interparticle contact and produced an island-like particle distribution, indicating that the green body could no longer maintain sufficient strength. In comparison, the specimens decarburized at 400 and 600 °C retained a relatively intact particle network, and interparticle gaps disappeared in some regions, suggesting the onset of neck formation between particles. These observations indicate that the oxidation behavior of MDLP-fabricated 316L SS green bodies differs markedly from that of dense 316L SS. Because the debound body consists of loosely packed particles with numerous open pores and a high specific surface area, oxygen can readily penetrate into the interior and react with the metallic skeleton, thereby lowering the temperature at which significant oxidation occurs. Based on these results, 400–600 °C was determined to be the appropriate temperature range for decarburization.

Overall, the results demonstrate that an effective residual-carbon removal strategy for MDLP-fabricated 316L SS consists of debinding in an inert atmosphere followed by decarburization in air at 400–600 °C. This route removes residual carbon efficiently while avoiding both debinding-induced cracking and oxidation-related damage to the green body.

### 3.2. Effect of Sintering Parameters on Microstructure and Mechanical Properties

**Figure 6:**
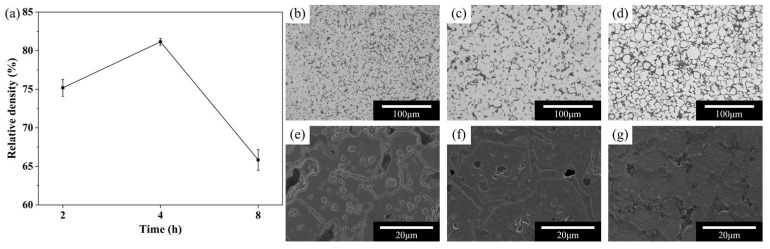
Effect of sintering holding time on the density and microstructure. (**a**) Relative density; (**b**–**g**) Optical micrograph (unetched) and SEM image (etched) of the 2 h, 4 h and 8 h group.

The effect of sintering holding time on densification and microstructural evolution is shown in Figure 6. As the holding time increased from 2 to 4 h, the relative density increased significantly, indicating enhanced densification. Correspondingly, the microstructure evolved from one containing numerous pores, many of which were open, to a more compact structure with fewer pores and clearer grain boundaries (as shown in Figure 6b,c,e,f). These observations suggest that extending the holding time from 2 to 4 h promoted neck growth and pore closure, thereby enhancing densification and microstructural integrity.

However, when the holding time was further increased to 8 h, the density decreased rather than continuing to increase. SEM observations showed that the particle contours became more apparent again, indicating deterioration in sintering quality (Figure 6d,g). One possible explanation for this unexpected decrease is that prolonged holding increased the likelihood of oxidation due to residual oxygen or slight air leakage in the furnace, which hindered further densification. Alternatively, excessive holding may have promoted pore coarsening or abnormal grain growth. Nevertheless, these interpretations remain tentative, as direct evidence of oxygen ingress or pore coarsening kinetics was not obtained in this study. Therefore, 4 h was selected as the optimal holding time for sintering.

**Figure 7:**
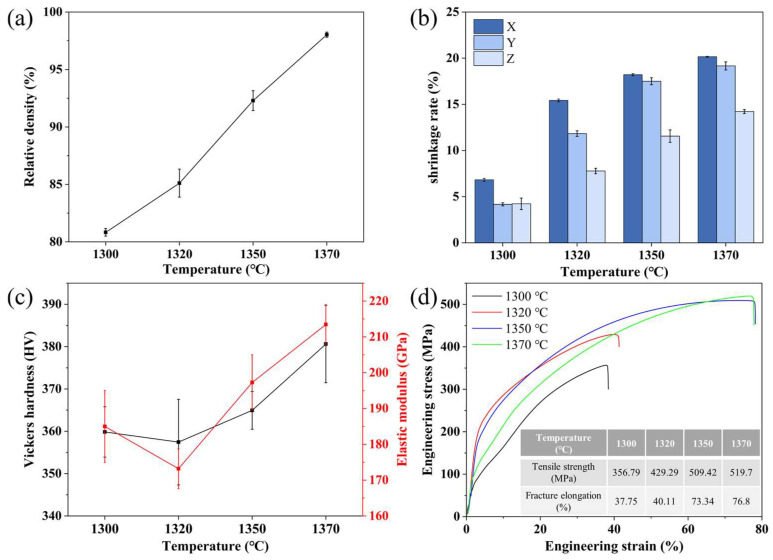
Effect of sintering temperature on the mechanical properties. (**a**) Relative density; (**b**) Linear shrinkage; (**c**) Hardness and elastic modulus; (**d**) Tensile strength and fracture elongation.

The effect of sintering temperature on densification, shrinkage, and mechanical properties is summarized in Figure 7. With increasing sintering temperature, the relative density increased continuously and reached 98.03 ± 0.23% at 1370 °C. The linear shrinkage showed a corresponding increase, consistent with progressive densification. The hardness and elastic modulus remained relatively low at 1300 and 1320 °C, but increased markedly from 1350 °C onward, reaching 380.63 ± 9.15 HV and 213.47 ± 5.5 GPa, respectively, at 1370 °C. The slight apparent decrease in hardness between 1300 and 1320 °C falls within the standard deviation of the measurements and likely reflects localized variations in residual porosity rather than a genuine material softening trend. A similar trend was observed for tensile strength and elongation at fracture, which increased substantially at higher temperatures and reached 519.7 ± 22 MPa and 76.8 ± 9.3%, respectively, at 1370 °C. These results indicate that increasing the sintering temperature within the investigated range substantially improved both densification and mechanical properties.

Although further increasing the sintering temperature would be expected to promote diffusion, preliminary experiments showed that melting occurred at 1380 °C. Accordingly, 1370 °C was selected as the optimal sintering temperature. Taken together, the results of the holding-time and temperature experiments indicate that sintering at 1370 °C for 4 h provides the best balance between densification, shape retention, and mechanical properties. The microstructural origin of these property changes is discussed in the following section.

### 3.3. Evolution of Oxygen-Rich Phases and Pores with Sintering Temperature and Their Influence on Sample Properties

**Figure 8:**
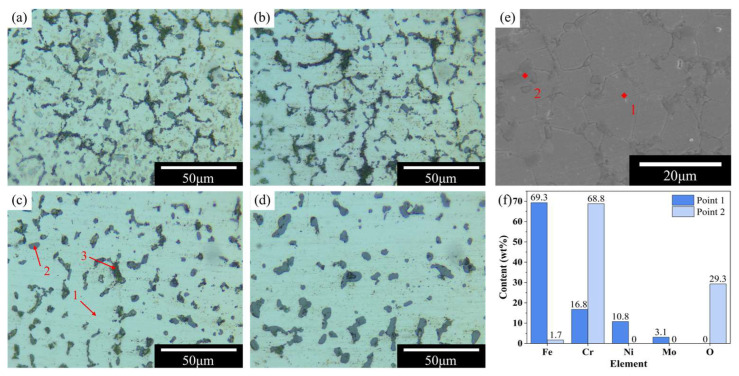
Effect of sintering temperature on the microstructure and elemental distribution of samples. (**a**–**d**) Microstructures sintered at 1300, 1320, 1350, 1370 °C; (**e**) SEM micrograph after etching; small squares and numbers 1 and 2 mark the EDS point analysis sites (1: matrix; 2: oxygen-rich second phase). (**f**) EDS elemental distribution corresponding to (**e**).

Figure 8 shows the microstructures of the samples sintered at different temperatures. Under optical microscopy, three characteristic features can be identified: the white 316L SS matrix (point 1), a black second phase (point 2), and pores (point 3) distributed between the matrix and the second phase. As the sintering temperature increased, both the number and area of pores decreased, consistent with the measured increases in density and shrinkage. At lower temperatures, the second phase was distributed along the original particle contours and exhibited a sharp-edged morphology. As the temperature increased, this phase became progressively more rounded and was increasingly confined to grain-boundary regions, as shown in Figure 8a–d. Elemental analysis further revealed that the second phase was enriched in oxygen and chromium relative to the metallic matrix, as shown in Figure 8e,f.

**Figure 9:**
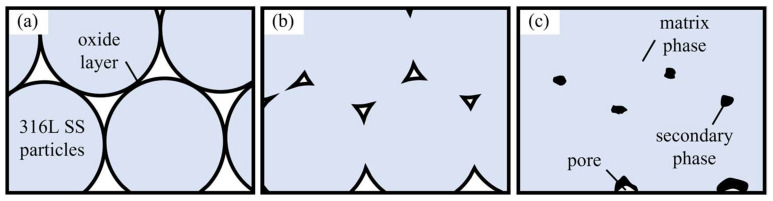
Schematic diagram of the microstructure evolution at different sintering temperatures. (**a**) Lower sintering temperature; (**b**) medium sintering temperature; (**c**) higher sintering temperature.

The above observations provide insight into the microstructural evolution during sintering. As shown in Figure 9a, the 316L SS particles were initially covered by a thin chromium-rich oxide layer formed during exposure to air. Because neither the debinding nor the sintering process involved a reducing atmosphere, and because most of the residual carbon was removed during decarburization, it is likely that this oxide layer was not fully reduced during thermal processing and therefore remained in the final microstructure as an oxygen-rich second phase. Similar retention of oxides has been reported in sintering studies of stainless steel powders processed under non-reducing atmospheres [27]. At low sintering temperatures, only limited neck growth occurred between adjacent particles, leaving large pores and sharp oxide-decorated particle boundaries, as illustrated in Figure 9b. As the temperature increased, diffusion became more active, the sintering necks expanded, and the metallic matrix became more continuous. Consequently, the pore volume decreased, the original particle contours gradually disappeared, and the second phase was squeezed into smoother grain-boundary distributions, as shown in Figure 9c.

This microstructural evolution provides a plausible explanation for the trends in mechanical properties shown in Figure 7. At 1300 and 1320 °C, the microstructure contained a large number of pores and sharp-edged second-phase regions. The pores reduced the effective load-bearing area and weakened the local particle skeleton, thereby lowering the hardness and elastic modulus. At the same time, it is reasonable to infer that the sharp morphology of the second phase likely acted as a stress concentration site during tensile deformation, which would facilitate crack initiation and contribute to the observed reduction in both tensile strength and elongation at fracture. In contrast, at 1350 and 1370 °C, the reduced porosity and smoother second-phase are expected to alleviate stress concentration and improve matrix continuity, thereby contributing to the substantially better mechanical properties. In addition, the grain-boundary distribution of the second phase may also have contributed to suppressing excessive grain growth, which was also beneficial to strength and ductility.

**Figure 10:**
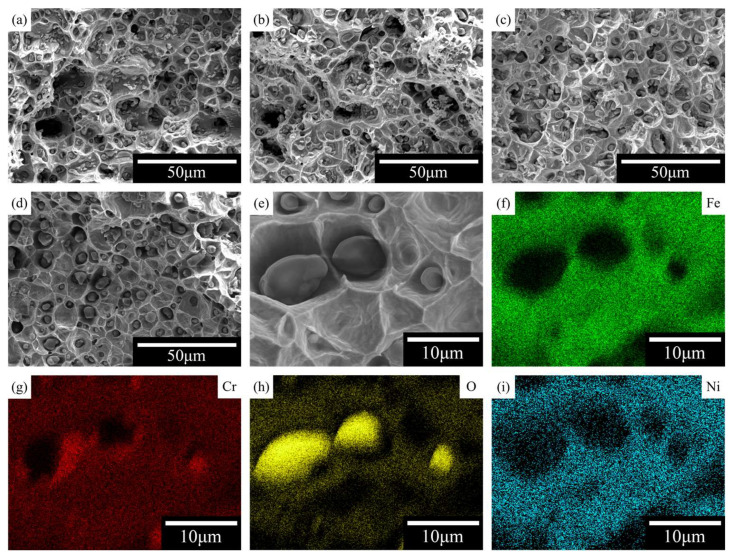
Effect of sintering temperature on tensile fracture morphology and elemental distribution on the fracture surface. (**a**–**d**) Fracture morphologies of samples sintered at 1300, 1320, 1350, 1370 °C; (**e**) Higher-magnification image of the fracture surface; (**f**–**i**) Elemental maps of Fe, Cr, O, Ni. All SEM images in panels (**a**–**e**) were acquired using secondary electron (SE) imaging mode to highlight topographic contrast.

The fracture-surface observations in Figure 10a–d further support this interpretation. All samples exhibited ductile fracture characteristics, with dimples observed on the fracture surfaces. However, the samples sintered at lower temperatures contained more voids, consistent with their lower tensile strength and fracture elongation. As the sintering temperature increased, the number and size of these voids decreased markedly. Moreover, EDS mapping of the inclusions located at the bottoms of the dimples showed strong enrichment in oxygen and chromium, whereas the surrounding matrix contained Fe, Cr, and Ni (Figure 10e–i). This suggests that the inclusions are associated with a Cr-rich oxygen-containing second phase, consistent with the second phase regions observed in the polished microstructures. Collectively, these observations indicate that the final mechanical behavior of MDLP-fabricated 316L SS is influenced not only by the degree of densification, but also by the morphology and distribution of oxygen-rich second phase regions formed during post-processing.

It is worth noting that the post-processing behavior of MDLP differs in several important respects from that of MIM and BJ. MDLP feedstocks typically contain a higher binder fraction (50 vol%) and employ photocurable resins that must be removed by thermal decomposition rather than solvent extraction. This combination results in a substantial amount of residual carbon after debinding, which necessitates a dedicated decarburization step prior to sintering. In contrast, MIM relies on a multi-step debinding process combining solvent and thermal stages, whereas BJ parts contain far less binder and can be processed via a continuous thermal debinding–sintering cycle with appropriate binder selection, thereby eliminating the need for an intermediate decarburization step [28,29,30,31]. These differences highlight the necessity of a dedicated post-processing route for MDLP-fabricated 316L SS.

In summary, the optimal post-processing route for MDLP-fabricated 316L SS consists of debinding in an inert atmosphere, decarburization in air at 400–600 °C, and sintering at 1370 °C for 4 h under flowing nitrogen. This route suppresses debinding-induced cracking, avoids oxidation-related damage during decarburization, eliminates melting associated with residual carbon, and yields nearly dense 316L SS parts with balanced microstructural and mechanical properties.

## 4. Conclusions

In this study, the post-processing route for MDLP-fabricated 316L stainless steel (316L SS) was systematically optimized by investigating the effects of debinding, decarburization, and sintering on densification, shape fidelity, microstructure, and mechanical properties. The key findings are summarized as follows:

- An optimal post-processing route was identified: debinding in an inert atmosphere, decarburization in air at 400–600 °C, and sintering at 1370 °C for 4 h under flowing nitrogen. Under these conditions, the sintered parts achieved a relative density of 98.03 ± 0.23%, a hardness of 380.63 ± 9.15 HV, an elastic modulus of 213.47 ± 5.5 GPa, a tensile strength of 519.7 ± 22 MPa, and an elongation at fracture of 76.8 ± 9.3%.
- Residual carbon must be removed prior to sintering to prevent melting-induced shape distortion, while excessive oxidation during decarburization must be avoided to preserve the structural integrity of the debound body.
- Increasing the sintering temperature was found to promote densification and was accompanied by a morphological transition of the Cr-rich oxygen-containing second phase from a sharp-edged morphology toward a smoother grain-boundary distribution. This transition is expected to alleviate stress concentration and thereby contribute to the observed improvement in mechanical properties.
- The final mechanical performance of MDLP-fabricated 316L SS is influenced not only by the degree of densification, but also by the morphology and distribution of oxygen-rich second-phase regions that evolve during post-processing.

Overall, this work establishes an effective post-processing strategy for MDLP-fabricated 316L SS and provides mechanistic insight into how residual carbon, oxygen-rich second phases, and pore evolution govern the final properties of the sintered parts.

## Figures and Tables

**Table 1:** Elemental composition of 316L SS powder (wt%).

Element	Fe	Ni	Cr	Mo	C	Mn	Si	P	S	O
Proportion	Bal	10.53	17.25	2.20	0.025	0.91	0.63	0.027	0.01	0.062

## Data Availability

The original contributions presented in this study are included in the article. Further inquiries can be directed to the corresponding authors.
